# Supplementary material for: Pre-gestational diabetes: Maternal body mass index and gestational weight gain are associated with augmented umbilical venous flow, fetal liver perfusion, and thus birthweight
Source: PLoS One. 2021 Aug 16;16(8):e0256171. doi: 10.1371/journal.pone.0256171 (PMC8367003; doi:10.1371/journal.pone.0256171)
Supplement: Attachment — Submitted filename: Response to Reviewers.docx [file pone.0256171.s001.docx]

Dear Academic Editor Umberto Simeoni.

We thank for the review of our paper (PONE-D-21-10502)
"Pre-gestational diabetes: maternal body mass index and gestational weight gain augments umbilical venous flow, fetal liver perfusion, and thus birthweigh). We appreciate the opportunity to improve our manuscript and to reply to comments.

We have discussed each point raised and carried out changes in the manuscript. Please find our responses below.

“Dear Dr. Ebbing,

Thank you for submitting your manuscript to PLOS ONE. After careful consideration, we feel that it has merit but does not fully meet PLOS ONE’s publication criteria as it currently stands. Therefore, we invite you to submit a revised version of the manuscript that addresses the points raised during the review process.
Please take into account all the remarks made by Reviewer 2 in your revised manuscript.

***Response:*** *Thank you. We will explain how the remarks has been accounted for in the revised manuscript.*

In addition, we note that the manuscript text contains numerous causal inferences, regarding both the hypothesis and the studied potential mechanisms. Although the study includes a comparison with a reference group, its observational design does not allow firm causative conclusions in our opinion, despite the likelihood and potential interest of the hypotheses. We would suggest that any terms like "effect", "impact", and other explicit causative wording be avoided and replaced by "associations". The potential mechanistic sequence described between the measured blood flows parameters and the birth weight should thus still be presented as an - eventually strong - hypothesis. These changes in the wording should be applied to the entire text, including the manuscript title and the abstract.

***Reply***: *Thank you! We carried out changes throughout the manuscript, and these are marked in the new version of the manuscript with Track Changes. The changes implied a revised title and conclusion in the abstract.*

**Title: “Pre-gestational diabetes: maternal body mass index and gestational weight gain are associated with augmented umbilical venous flow, fetal liver perfusion, and thus birthweight.”**

Revised conclusions abstract:” The results support the hypothesis that umbilical flow to the fetal liver is a key determinant for fetal growth and birthweight modifiable by maternal factors. Maternal pre-gestational diabetes mellitus seems to augment this influence as shown with body mass index and gestational weight gain.”

1. Please ensure that your manuscript meets PLOS ONE's style requirements, including those for file naming. The PLOS ONE style templates can be found at

<https://journals.plos.org/plosone/s/file?id=wjVg/PLOSOne_formatting_sample_main_body.pdf> and

https://journals.plos.org/plosone/s/file?id=ba62/PLOSOne_formatting_sample_title_authors_affiliations.pdf

***Reply***: *We have made sure that our manuscript meets PLOS one style requirements.*

2. Please review your reference list to ensure that it is complete and correct. If you have cited papers that have been retracted, please include the rationale for doing so in the manuscript text, or remove these references and replace them with relevant current references. Any changes to the reference list should be mentioned in the rebuttal letter that accompanies your revised manuscript. If you need to cite a retracted article, indicate the article’s retracted status in the References list and also include a citation and full reference for the retraction notice.
 ***Response:*** *We have identified a typo and added some references to the list in line with the changes we have carried out in response to the reviewer's comments.*

*3.* Please provide additional details regarding participant consent. In the ethics statement in the Methods and online submission information, please ensure that you have specified whether consent was informed.

***Reply***: *We have specified in the Methods section that participants had informed consent.*

*4. We note that you have indicated that data from this study are available upon request. PLOS only allows data to be available upon request if there are legal or ethical restrictions on sharing data publicly. For information on unacceptable data access restrictions, please see* [*http://journals.plos.org/plosone/s/data-availability#loc-unacceptable-data-access-restrictions*](http://journals.plos.org/plosone/s/data-availability#loc-unacceptable-data-access-restrictions)*.*

*a) If there are ethical or legal restrictions on sharing a de-identified data set, please explain them in detail (e.g., data contain potentially identifying or sensitive patient information) and who has imposed them (e.g., an ethics committee). Please also provide contact information for a data access committee, ethics committee, or other institutional body to which data requests may be sent.*

*b) If there are no restrictions, please upload the minimal anonymized data set necessary to replicate your study findings as either Supporting Information files or to a stable, public repository and provide us with the relevant URLs, DOIs, or accession numbers. Please see* [*http://www.bmj.com/content/340/bmj.c181.long*](http://www.bmj.com/content/340/bmj.c181.long) *for guidelines on how to de-identify and prepare clinical data for publication. For a list of acceptable repositories, please see* [*http://journals.plos.org/plosone/s/data-availability#loc-recommended-repositories*](http://journals.plos.org/plosone/s/data-availability#loc-recommended-repositories)*.*

*We will update your Data Availability statement on your behalf to reflect the information you provide.*

***Reply***: *Please see the revised cover letter.*

Reviewers' comments:

Reviewer's Responses to Questions

**Comments to the Author**

**1. Is the manuscript technically sound, and do the data support the conclusions?**

**The manuscript must describe a technically sound piece of scientific research with data that supports the conclusions. Experiments must have been conducted rigorously, with appropriate controls, replication, and sample sizes. The conclusions must be drawn appropriately based on the data presented.**

Reviewer #1: Yes

Reviewer #2: Yes

2. Has the statistical analysis been performed appropriately and rigorously?

Reviewer #1: Yes

Reviewer #2: Yes

3. Have the authors made all data underlying the findings in their manuscript fully available?

The [PLOS Data policy](http://www.plosone.org/static/policies.action#sharing) requires authors to make all data underlying the findings described in their manuscript fully available without restriction, with rare exception (please refer to the Data Availability Statement in the manuscript PDF file). The data should be provided as part of the manuscript or its supporting information, or deposited to a public repository. For example, in addition to summary statistics, the data points behind means, medians and variance measures should be available. If there are restrictions on publicly sharing data—e.g. participant privacy or use of data from a third party—those must be specified.

Reviewer #1: Yes

Reviewer #2: Yes

4. Is the manuscript presented in an intelligible fashion and written in standard English?

PLOS ONE does not copyedit accepted manuscripts, so the language in submitted articles must be clear, correct, and unambiguous. Any typographical or grammatical errors should be corrected at revision, so please note any specific errors here.

Reviewer #1: Yes

Reviewer #2: Yes

5. Review Comments to the Author

Reviewer #1: The Authors in this prospective longitudinal observational study acess ,the modifying effects of maternal BMI and gestational weight gain ,on the venous perfusion of the fetal liver and birthweight. In this work they investigate fetal hemodynamics of liver perfusion evaluating the development of ductus venous, umbilical and portal blood flows during 2nd trimester of pregnancies with PGDM.
 The results are very original and sound, explaning larger birthweights of fetuses in those pregnancies as part of perinatal programming of future cardiovascular risk of newborns of those pregnancies.

Reviewer #2: In their longituonal observational study in women with pregestational DM the authors identify umbilical venous bloodflow to be significantly associated with birthweight. Importantly, this association is augmented in women with PGDM when compared to appropriately selected controls.
 The vast majority (90%) of women in the PGDM have T1DM with on average fairly good metabolic control (HbA1c 6.7%).
The authors report that the effects of maternal weight and weight gain on birthweight are associated with umbilical blood flow but not metabolic control, as indicated by HbA1c. It would be interesting to know, whether metabolic control per se impacts umbilical blood flow.
 Although still considered the most important surrogate of glycemic control, HbA1c has several limitations in pregnancy. Can the authors present data or at least speculate whether glycemia per se, i.e. ambient glucose levels and/or glycemic variability affect umbilical venous blood flow and whether this could have affected their results.

***Reply****: Thank you for this comment. Unfortunately, we do not have access to ambient glucose levels or variability in our study population at the time of ultrasound and Doppler evaluation of the UV flow. We have explored the association between maternal glycemic control and fetal venous liver flow in two previous publications (Lund A et al Acta Obstet Gynecol 2018 Aug;97(8):1032-1040; Lund A. et al, PloS One 2019; 14(3), e0211788). We found that there was a negative relation between maternal HbA1c and the ductus venosus flow velocity, flow volume and shunt fraction, especially near term. Further, maternal HbA1c had a positive association to left portal vein flow velocities and a negative relation to the contribution of portal blood flow to the venous liver flow. Fetuses of women with high HbA1c had higher umbilical blood flow, this association was however not statistically significant (Fig. 1). We may speculate that in women with HbA1c levels that are low, shunt fraction and UV flow resembles the distribution in women without diabetes.*

***Fig. 1:*** *Umbilical blood flow according to maternal 1^st^ trimester HbA1c in PGDM*

The authors discuss a possible role of maternal prandial status on umbilical blood flow. Was this considered when the experiments were performed ?

***Reply****: Unfortunately, we did not register the timing or composition of the last meal in relation to the Doppler/ultrasound evaluation. We realize in hindsight that this would have been an interesting aspect. The women did not seldomly check their sugar levels during examination, and some even had a snack on the examination bench during examination when glucose levels were low.*

The authors speculate on a possible association of hypoglycemic episodes, gestational weight gain, altered fuel supply, i.e. increased lipid/FFA delivery to the fetus. However, repeat hypoglycemia has been associated with fetal growth restriction. Please discuss.

***Reply***: *Thank you for this important comment. We agree, excessive gestational weight gain is independently associated with increased risk of large for gestational age neonates, and hypoglycemia is associated with low birthweight and placental weights. Excessive GWG is associated with hypertension. Altered fuel supply to the fetus as a response to hypoglycemia may be influenced by maternal factors such as duration of the disease, responder status and complications (nephropathy, retinopathy). There is a clinical impression that frequent hypoglycemic events lead to extra carbohydrate intake which again may add to increased gestational weight gain. The present study does not contain data on nutritional factors (lipids and fasting glucose levels) and cannot answer the question whether episodes of hypoglycemia alter the substrate supply to the fetus, but this should be explored. We have now elaborated on this and adjusted the discussion.*

“ Further, defect epinephrine counter-regulation during hypoglycemia in PGDM pregnancies contributes to excessive fetal growth [33], probably through compensatory bouts of calorie intake with subsequent fetal hyperinsulinemia.

In women with PGDM, gestational weight gain contributes to excessive fetal growth, independent of maternal BMI and glycemic control [8, 34]. The mechanisms are not completely understood, but additional nutrients delivery (fatty acids and amino acids) and altered leptine levels are suggested to contribute to accelerated fetal growth [35,36]. In addition to nutritional and hormonal influence, the present study suggests fetal blood flow as a possible link between maternal GWG and increased birth weight”

The authors report that several patients suffered from chronic hypertension. Could the maternal intake of antihypertensives, presumably mostly betablockers, have influenced the results of the study ?

***Reply***: *Absolutely, we agree that we cannot rule out that the use of antihypertensive medicaments may influence UV flow. In our study population seven of the participants had chronic hypertension. We did not perform any sub-analyses in this group due to the small size of the population. We acknowledge that maternal use of antihypertensives may alter fetal distribution of umbilical flow, however, human evidence is scarce, and evidence in pregnancies with DM1 are lacking. Jouppila,P. et al have performed studies on antihypertensives and the effect on fetal and maternal hemodynamics.* *Their findings in women and fetuses with preeclampsia suggested that labetalol reduces maternal blood pressure without interfering with the placental or fetal blood flow (Jouppila P et al Br J Obstet Gynecol 1986 Jun;93(6):543-7). While evidence from experimental studies in sheep showed that labetalol reduced placental volume flow, and increased placental vascular resistance. (Erkinaro T et al Reprod Sci 2009* *Aug;16(8):749-57).*  *We have now inserted a sentence in the discussion section about this issue.*

“...study. Seven women in the study population used anti-hypertensive drugs which may influence maternal and feto-placental hemodynamics [52, 53]. We considered the size of the study population too small for subgroup analyses of maternal ethnicity, the use of antihypertensive drugs or sex of the neonate.”

6. PLOS authors have the option to publish the peer review history of their article ([what does this mean?](https://journals.plos.org/plosone/s/editorial-and-peer-review-process#loc-peer-review-history)). If published, this will include your full peer review and any attached files.


If you choose “no”, your identity will remain anonymous but your review may still be made public.


Do you want your identity to be public for this peer review? For information about this choice, including consent withdrawal, please see our [Privacy Policy](https://www.plos.org/privacy-policy).

Reviewer #1: No

Reviewer #2: No

[NOTE: If reviewer comments were submitted as an attachment file, they will be attached to this email and accessible via the submission site. Please log into your account, locate the manuscript record, and check for the action link "View Attachments". If this link does not appear, there are no attachment files.]

While revising your submission, please upload your figure files to the Preflight Analysis and Conversion Engine (PACE) digital diagnostic tool, <https://pacev2.apexcovantage.com/>. PACE helps ensure that figures meet PLOS requirements. To use PACE, you must first register as a user. Registration is free. Then, login and navigate to the UPLOAD tab, where you will find detailed instructions on how to use the tool. If you encounter any issues or have any questions when using PACE, please email PLOS at [figures@plos.org](mailto:figures@plos.org). Please note that Supporting Information files do not need this step.

***Response:*** *All figure files were approved in PACE*
